# Replacement of Vitamin E by an Extract from an Olive Oil By-Product, Rich in Hydroxytyrosol, in Broiler Diets: Effects on Growth Performance and Breast Meat Quality

**DOI:** 10.3390/antiox12111940

**Published:** 2023-10-31

**Authors:** Nereida L. Corrales, Fernando Sevillano, Rosa Escudero, Gonzalo G. Mateos, David Menoyo

**Affiliations:** 1 Departamento Producción Agraria, Escuela Técnica Superior de Ingeniería Agronómica, Alimentaria y de Biosistemas, Universidad Politécnica de Madrid, Avda. Puerta de Hierro 2, 28040 Madrid, Spain; nereida.lunac@alumnos.upm.es (N.L.C.); f.sevillano@alumnos.upm.es (F.S.); gonzalo.gmateos@upm.es (G.G.M.); 2 Departamento Producción Animal, Facultad de Veterinaria, Universidad Complutense de Madrid, Avda. Puerta de Hierro s/n, 28040 Madrid, Spain; rmescude@ucm.es

**Keywords:** broiler growth, hydroxytyrosol, malonaldehyde, olive oil by-product, vitamin E

## Abstract

The hypothesis of this experiment was that a liquid rich in hydroxytyrosol (HT) obtained from “alperujo”, an olive oil by-product, could replace part of the added vitamin E (VE) as an antioxidant in poultry diets. There were five diets that differed exclusively in the substitution of supplemental VE (0 to 40 mg/kg, with differences of 10 mg/kg) by HT (30 to 0 mg/kg, with differences of 7.5 mg/kg). The basal diet was based on corn and soybean meal and provided 10 mg VE/kg. From 0 to 39 d of age, the growth performance of the birds was not affected by diet. The birds were slaughtered at 39 d of age to evaluate the quality of the breast, and malonaldehyde concentration, pH, color, and drip loss were measured. In terms of meat lipid oxidation, the combination of 22.5 mg HT/kg and 10 mg of added VE/kg equalized to a diet supplemented with 40 mg VE/kg. Meat color improved in broilers fed 7.5 mg HT/kg and 30 mg VE/kg. It is concluded that once the nutritional requirements of the birds in VE are satisfied, the dietary supplementation with the olive oil by-product rich in HT can be used as a strategy to spare VE in broiler diets.

## 1. Introduction

Broiler meat production is more efficient and cost-effective now than 20 years ago because of advances in genetic selection, improvements in management, and changes in nutritional practices. Genetic selection programs, based on greater feed intake, faster growth rates, and greater breast yield, have led to a higher metabolic rate and an increase in the exposure of the birds to oxidative stress [1,2]. In addition, poultry meat is rich in unsaturated fatty acids, which are more susceptible to be oxidized during processing and storage [2]. Therefore, it is important to boost the antioxidant defenses of the animal by adding to the diet additives and nutrients with a high antioxidant capacity, such as α-tocopherol, ascorbate, selenium, magnesium, and zinc [1]. The antioxidant capacity has been well studied in the last years, with vitamin E (VE) being the most effective and used additive in animal nutrition with this role [3]. According to the National Research Council (NRC), the minimum level of VE in the diet to meet the nutritional requirements, avoiding any physiological deficiencies in poultry, is 10 IU/kg [4]. However, the basal requirement of VE varies with the physiological state of the bird, and the amount of VE added is compromised by the high variability in its concentration for most ingredients used in practical diets [5]. In this respect, the recommendations of the Fundación Española Para la Nutrición Animal (FEDNA) on VE in practical diets for broilers range between 25 and 50 mg/kg, highlighting that the level of use can be increased by the inclusion of extra VE (or any other antioxidant), according to production objectives [6]. In fact, an increase in VE supplementation of the diet above physiological levels (e.g., 50 to 100 mg/kg) might add extra benefits to the birds by improving the immune response against oxidative stress, resulting in better growth performance under common stress situations. In this respect, an increase in dietary VE (200 mg/kg) might prevent lipid rancidity by improving meat quality, increasing meat shelf life [7]. Consequently, many poultry integrators recommend adding more than the standard levels of VE in broiler diets despite increases in price [8,9]. The high variability on the levels of use of VE in commercial diets for broilers has been recently highlighted in a study comparing feeding programs and premix composition by the Spanish poultry industry [10]. Because of economic reasons and the synthetic nature of commercial VE, researchers are looking for more cost-effective and natural alternatives. In this respect, the research on phytogenic feed additives with antioxidant capacity has notably increased for the last decade [1].

The use of agro-industrial by-products is a key strategy to reduce feeding costs in animal feeding and to cope with the need to recycle waste material [11]. The production of olives (*Olea europaea*) and the olive oil industry play a significant socio-economic role in many Mediterranean countries, with around 1.2 million tons of olive oil per year, with Spain being the largest producer worldwide [12,13]. Nowadays, the process of oil extraction from the olives in Spain is carried out predominantly by the two-centrifugation system, which generates a final waste product referred to as “alperujo” or olive pomace. This high moisture waste is characterized by its high organic matter content, which is composed mainly of fiber, fats, proteins, water-soluble carbohydrates, and phenolic substances [14]. The percentage of oil extracted from the olives fluctuates between 10 and 25%, and thus, Spain generates between 0.9 and 1.08 million tons of olive waste in addition to the 0.5 L of extra water required per kg of olives, which results directly from olive processing [15]. An opportunity to take advantage of wastes from the olive industry is its use in animal feeds. “Alperujo”, the olive by-product most frequently used in animal feed, has shown satisfactory results when fed directly at low doses [16,17,18]. Furthermore, because of its high content in bioactive compounds, phenolic substances are generally extracted and used in animal diets with a great interest for the feed and food industry [19,20,21].

Olives contain numerous polyphenolic compounds with high nutritional and economical values [22]. During the oil extraction process, most of the phenolic compounds presents in the olives, such as hydroxytyrosol (HT), remain in the wastewater and the pomace [23,24,25]. Many of the olive oil by-products have been associated with antioxidant, anti-inflammatory, antimicrobial, and anti-coccidian effects in poultry [18,26,27], with HT showing the strongest antioxidant activity among the various existing phenols. In this respect, the high antioxidant efficiency of HT is due to the presence of hydroxyl groups which act as chain breakers by donating a hydrogen atom to peroxyl-radicals [28]. Many studies have shown that the inclusion in the diet of natural antioxidant compounds, such as those present in olive oil by-products, improves the oxidative stability of chicken meat [26,29,30]. Moreover, Balzan et al. [31] reported the presence of HT in the breast of broilers fed diets supplemented with phenolic concentrate from olive vegetation water, showing that the absorption of this compound by the chicken is quite effective. All this information indicates that the inclusion of olive oil by-products in the diet could be a sound strategy to reduce the oxidative deterioration of broiler meat. The hypothesis postulated in the current research is that a liquid rich in HT and obtained from “alperujo” can replace part of the VE currently used as an antioxidant in commercial diets for broilers without showing any negative effect on growth performance or meat quality. 

## 2. Materials and Methods

### 2.1. Birds and Husbandry

The feeding trial was approved by the Ethics Committee of the Universidad Politécnica de Madrid (Permission no: 2021-001). All the experimental procedures and animal care followed the established Spanish Guidelines for the Care and Use of Animals in Research [32].

In total, 560 one-day-old, male Cobb-500 broilers were obtained from a commercial hatchery. The chicks (initial BW of 41.3 ± 0.66 g) were randomly allotted to 35 floor pens in an environmentally controlled room. The pens (0.96 m × 1.50 m) were bedded with cereal straw, previously pelleted and rolled, and were provided with a hopper feeder and 4 bell drinkers. The environmental conditions of the barn during the experiment were controlled automatically, and bird management was as recommended for broilers under commercial conditions [33]. Chicks had free access to feed and water throughout the experiment. The temperature was set at 33 °C during the first week of age and then was reduced 2 °C per week until reaching 21 °C at 21 d of age, maintaining this temperature until the end of the trial. The broilers received a light program of 23 h light from 1 to 7 d of age and 18 h light until the end of the experiment. 

### 2.2. Diets and Experimental Design

The experimental product tested was OLIVOX^®^ (provided by Nutrición y Gestión S.L., Badajoz, Spain) obtained from the olive waste “alperujo” during the olive oil extraction process using a patented industrial procedure (ES2436626B1). The process consists of the mechanical separation of the “alperujo” by decantation into two phases: liquid and solid. The solid fraction is characterized by its high content in neutral detergent fiber and in fat, which is rich in oleic acid. The liquid fraction (OLIVOX^®^) contains 53.2% of moisture and 18.2% of ash and has a high concentration of polyphenols (an average of 23.6 mg equivalents of gallic acid per g of product). Prior to its use as an ingredient in the feed, the liquid fraction was absorbed in a silica carrier (SIPERNAT, Evonik Corporation, Parsippany, NJ, USA) in proportions of 60 and 40%, respectively. The levels of HT established in this research were based on the concentrations used by other researchers who observed positive effects on the performance and oxidative stability of meat when included at 9.5 mg HT per kg of feed [34]. The final mixture (OLIVOX^®^ in a silica carrier) was included in the experimental feeds to ensure the amount of desired HT (0, 7.5, 15, 22.5 and 30 mg of HT per kg of feed).

The experiment was conducted as a completely randomized design with 5 treatments (HT0, HT7.5, HT15, HT22.5 and HT30) that consisted of 5 levels of HT (from 30 to 0 mg HT/kg), with a difference of 7.5 mg/kg between diets, used in the substitution of VE (from 0 to 40 mg VE/kg) with a difference of 10 mg/kg between diets. The feeding program consisted of two phases: starter (0 to 21 d) and grower (22 to 39 d). Each diet was replicated 7 times, and the experimental unit was a floor pen with 16 chicks. The diets were based on corn and soybean meal (SBM) and were fed in crumble and pellet form. The starter and grower diets that did not include any supplemental VE contained 13.7 and 14.5 mg/kg of basal VE, respectively. All diets were formulated to meet the nutrient requirements of the broilers [6] and were manufactured at the Institute of Agrifood Research and Technology (IRTA; Mas de Bover, Constanti, Spain). 

### 2.3. Laboratory Analysis

Representative samples of the diets were ground using a hammer mill (Model Z-I, Retsch, Stuttgart, Germany), fitted with a 0.75 mm screen and analyzed for moisture by oven-drying (method 930.15), total ash with a muffle furnace (method 942.05), and nitrogen (N) by Kjeldahl (method 988.05) [35]. Crude protein content was calculated as N × 6.25. The gross energy of the experimental diets was determined using an adiabatic bomb calorimeter (model 1356, Parr Instrument Company, Moline, IL, USA), and VE was determined by HPLC [36]. All the analyses were conducted in triplicate.

The polyphenolic compounds were extracted from the OLIVOX^®^ and silica combination [37], and the total content was determined as indicated by the Folin–Ciocalteu method [38]. In addition, the HT and tyrosol contents were determined by HPLC [37]. The concentration of total polyphenols was 16.4 mg equivalents of gallic acid per g of product, and the HT and tyrosol contents were 9.10 and 2.55 mg per g of product, respectively, meaning around 71% of total polyphenols. The ingredient composition and calculated and determined analysis of the starter and grower experimental diets are shown in Table 1 and Table 2, respectively.

### 2.4. Measurements

The body weight (BW) and feed disappearance of the birds were recorded by replicate at 7, 14, 21, and 39 d of age, and mortality was recorded and weighted as produced. From these data, the average daily gain (ADG), average daily feed intake (ADFI), and feed conversion ratio (FCR) were determined by period (0 to 7, 8 to 14, 15 to 21, and 22 to 39 d of age), feeding phase (0 to 21 and 22 to 39 d of age), and cumulatively (0 to 39 d of age). 

At the end of the experiment, one bird per pen was randomly selected and slaughtered after 6 h of feed withdrawal period for meat quality analysis. The birds were weighed, euthanized by CO_2_ asphyxiation, exsanguinated, and chilled by immersion in water at 1 °C for 3 h. Then, the right *pectoralis major* was sampled and maintained for 2 h on ice for reducing the temperature. Later, it was stored at 4 °C, and samples were taken for meat quality analysis. Lipid oxidation was determined after 1, 3, and 7 d of storage at 4 °C using the 2-thiobarbituric acid method [39], which quantifies malonaldehyde (MDA) concentration. In addition, the pH was measured using a digital pH meter fitted with a fine tip glass electrode (model 507, Crison Instruments S.A., Barcelona, Spain). The average value of three independent measurements was used for further analysis. Breast color (lightness (L* values), redness (a* values), and yellowness (b* values)) was measured using a reflectance colorimeter (Minolta Chroma Meter CR-300, Minolta Italia S.p.A., Milano, Italy) after 1, 3, and 7 d of storage. The drip loss percentage was determined after 24 h of storage of the breast sample (weighting approximately 14 g) and calculated as the difference between the final and initial weight. During that time, the samples were suspended in a bag at 4 °C.

### 2.5. Statistical Analysis

The normal distribution of the residuals and the homogeneity of the variance of the data were tested using the UNIVARIATE procedure and Levene’s test, respectively (SAS Institute Inc., Cary, NC, USA). Data on growth performance and meat quality were analyzed as a completely randomized design with 5 treatments using the MIXED procedure of SAS (SAS Institute Inc., Cary, NC, USA) with the replacement of VE by HT in the diet as a fixed effect. The effects of the interaction between broiler age and the replacement of VE by HT in the diet on growth performance were tested. In addition, the effects of the length of the storage and the interaction between length and the replacement of VE by HT in the diet, on breast meat lipid oxidation and color, were also determined [40]. Mortality was analyzed using the GENMOD procedure of SAS. Regression analyses were used to measure the linear (L) and quadratic (Q) response to the replacement of VE by HT in the diet. When significant differences were detected, the Tukey test was used to separate treatment means. All differences were considered significant at *p* < 0.05, and values between 0.05 and 0.10 were considered a trend.

## 3. Results

### 3.1. Growth Performance

Mortality was 5.71% as an average and was not related to any treatment. From 0 to 39 d of age, no significant differences among treatments were detected for any of the variables studied (Table 3). The interaction between broiler age and the replacement of VE by HT in the diet was significant for ADFI (*p* = 0.041) due to an increase in ADFI during the last period in broilers fed diets including the experimental product. From 0 to 21 d of age, no significant differences were detected for any of the variables studied. From 0 to 7 d of age, however, an increase in HT content in the diet improved (L, *p* < 0.001; Q, *p* = 0.002) FCR, with the best result obtained with a combination of 22.5 mg HT/kg and 10 mg VE/kg. Similarly, ADG tended to increase as the content of HT in the diet increased (L, *p* = 0.053; Q, *p* = 0.076). From 22 to 39 d of age, ADFI increased as the HT content of the diet increased (L, *p* = 0.019; Q, *p* = 0.055). Moreover, significant differences on ADFI were detected between diets containing HT and the diet that did not contain the experimental product (*p* = 0.024), but ADG and FCR were not affected. 

### 3.2. Meat Quality

The effects of the replacement of VE by HT in the diet on breast meat lipid oxidation, color, pH, and drip loss are shown in Table 4. The concentration of MDA in the breast increased with storage time (*p* < 0.001). However, the rate of increase depended on the VE to HT ratio of the combinations (*p* < 0.001). After one day of storage, the concentration of MDA in the breast did not differ among treatments, but after 3 and 7 d of storage, the concentrations were higher (*p* < 0.05) in the breast of birds fed the HT30 diet. When the data were analyzed by regression, the level of VE of the diet did not affect MDA in the breast after 3 d of storage. However, after 7 d of storage, the quadratic effect was significant with MDA concentrations peaking in of birds fed the HT30 diet (L, *p* = 0.483; Q, *p* = 0.038). In relation to the color, significant differences were detected for lightness after one day of storage (L, *p* = 0.077; Q, *p* = 0.010) with broilers fed the diets HT30 and HT7.5 showing the highest and lowest values, respectively. However, no significant differences were detected at 3 and 7 d of storage. In contrast, no significant differences were detected for redness after one day of storage, but the values were higher at 3 and 7 d of storage for broilers fed the HT7.5 diet than for any of treatments (*p* < 0.01). Yellowness was not affected by treatment. The length of storage affected the lightness and yellowness of the breast (*p* < 0.001); however, no significant interactions between the replacement of VE by HT in the diet and the storage time were detected for the color of the breast. The drip loss and pH of the breast meat were not affected by dietary treatment. 

## 4. Discussion

### 4.1. Growth Performance

The replacement of VE by HT in the diet did not affect the growth performance of broilers from 0 to 39 d of age, which corroborates that under optimal conditions, an extra supplementation with VE, once the nutritional requirements on the vitamin are met, does not improve growth performance [41]. Under commercial conditions, however, higher levels of VE might be recommended to reinforce the antioxidant system of the bird against the oxidative stress caused by a variety of common stressors, including poor management (e.g., increased stocking density), extreme environmental conditions (e.g., heat stress), nutritional causes (e.g., oxidized fat), or physiological status (e.g., disbacteriosis at the gastrointestinal tract (GIT)), which might decrease the growth performance of the birds, causing important economic losses [42]. The results of the current study show that 30 mg HT/kg, provided by the olive oil by-product tested, can substitute up to 40 mg VE/kg without affecting bird performance. In research conducted by Sarica et al. [43], Japanese quails fed diets supplemented with 150 mg oleuropein/kg and a control diet with 200 mg VE/kg showed similar performance. In fact, in this study, the diet containing oleuropein was more effective in protecting polyunsaturated fatty acids from oxidation than the diets supplemented with VE. Consequently, it is plausive that olive oil by-products, rich in polyphenols, might spare VE in broiler feeds. However, further studies are needed to study this equivalence under practical situations.

In the present study, no differences in growth performance were detected for any of the variables studied during the starter period (0 to 21 d). However, from 0 to 7 d of age, an increase in HT content improved feed efficiency and tended to increase ADG. Moreover, from 22 to 39 d of age, ADFI increased as the level of HT in the diet increased, but ADG and FCR were not affected. Branciari et al. [34] reported also that the inclusion in the diet of a semi-solid olive cake, high in HT, increased ADG and improved FCR in broilers, which is an effect that increased as the level of HT in the diet increased. Herrero-Encinas et al. [27] observed that the supplementation of the diet fed from 22 to 42 d of age with olive extracts, rich in triterpenes and polyphenols, improved ADG and FCR but did not affect ADFI. In contrast, the addition of 0.5% of an olive leaf extract (equalizing 38.4 mg HT per kg of feed) reduced significantly the feed intake of broilers from 1 to 35 d of age, which was probably caused by the low palatability of the oleuropein used [44]. Similarly, Varmaghany et al. [45] reported that the addition of 15 g of olive leaves per kg of feed reduced the feed intake in broilers. Supplementation of the diets with phytogenics might increase nutrient digestibility by increasing digestive enzyme secretion and improving epithelial integrity, bile acid secretion, GIT motility and the microbiota profile of the cecum [46,47,48,49,50,51]. Moreover, the maturation of the intestinal mucosa as well as the growth of the main components of the GIT occur during the first 10 d of age [52], and consequently, the positive effects of the polyphenols may be more noticeable in young birds. It is hypothesized that the beneficial effects of HT supplementation reported in the current research were a consequence of the improvement in the oxidative state of the birds [26,44,47].

### 4.2. Meat Quality

In the current study, the combination of 22.5 mg HT/kg and 10 mg VE/kg was equal in terms of meat lipid oxidation to the inclusion in the diet of 40 mg VE/kg. However, after 3 and 7 d of storage, the highest MDA content in breast meat was observed in broilers fed the diet that did not include any extra VE. Numerous studies have shown that polyphenolic compounds present in olive oil by-products inhibit lipid peroxidation in poultry meat [26,34,44]. Even more, Balzan et al. [31] reported the presence of HT in the breast muscle of broilers, which indicates that HT is absorbed and metabolized efficiently in birds. Lipid peroxidation begins immediately after slaughter with a rate of progress that depends on the level of VE in the muscle, which in turn depends on the concentration of the vitamin in the diet [41]. Vitamin E plays an important role as a chain-breaking antioxidant, inhibiting the production of reactive oxygen species. The vitamin is located in the membranes of the cells and organelles, protecting them from damages caused by free radicals [53]. Because of its peroxyl radical scavenging activity, VE also protects the polyunsaturated fatty acids present in the phospholipids of the membrane [54]. On the other hand, HT has a high capacity to reduce or even eliminate the production of reactive oxygen species at intracellular and extracellular levels [55]. In addition, it has been shown that lipid-soluble and water-soluble antioxidants, such as VE and polyphenols, may act synergistically to control oxidation [56]. This redox cooperation takes place at the lipid–water interfaces in which the phenolic compounds might eliminate the oxidized tocopheroxyl radical produced in the VE peroxidation process, regenerating the vitamin and restoring its antioxidant function [56,57]. The data of the current study showed that the supplementation of the diet with 22.5 mg HT/kg combined with 10 mg VE/kg prevented breast meat lipid oxidation after 7 d of storage at 4 °C with results that were comparable to those observed when the broilers were fed a diet with 40 mg VE/kg. In a companion article of this special issue, we reported a linear decrease (*p* < 0.05) in α-tocopherol concentration in the liver with the replacement of VE by HT. Despite this VE reduction, no significant effects on the relative liver weight, total lipid, cholesterol, triglycerides, or TBARS concentrations were observed [58]. Therefore, either directly or through a synergistic mechanism, low inclusion levels of HT might be useful to spare VE or optimize the activity of the vitamin of the diet.

The heme pigments concentration in the muscles largely influences the appearance of poultry meat [59]. In fact, the heme content in the breast muscle is related positively to redness and negatively to lightness [60]. After slaughtering, breast meat maturation occurs along autoxidation processes, affecting the heme pigments [61]. Broilers fed the HT7.5 diet showed the lowest value for lightness and the highest for redness of the breast muscle. In this respect, the combination of 7.5 mg HT/kg and 30 mg VE/kg seems to inhibit heme autoxidation, thereby reducing the brightness and improving the redness values of the breast muscle. As previously indicated, a synergic effect between both antioxidants at low concentration in the diet might be responsible for this positive effect. The drip loss and pH of the breast meat were not affected by the diet, which is in agreement with the data of Branciari et al. [34] that indicated no changes in pH or drip loss of the breast meat of broilers fed 9.5 mg HT/kg provided by olive cake.

## 5. Conclusions

It is concluded that under the experimental conditions of this research, a combination of 22.5 mg HT/kg and 10 mg VE/kg equalizes to a diet supplemented with 40 mg VE/kg in terms of performance and meat lipid oxidation. 

## Figures and Tables

**Table 1:** Ingredient composition and calculated and determined analysis (% as feed basis) of the starter diets (0 to 21 d of age).

	HT0	HT7.5	HT15	HT22.5	HT30
**Ingredient**					
Corn	58.7	58.7	58.7	58.7	58.7
Soybean meal, 47% CP	36.4	36.4	36.4	36.4	36.4
Soy oil	1.20	1.20	1.20	1.20	1.20
Calcium carbonate	1.06	1.06	1.06	1.06	1.06
Monocalcium phosphate	1.02	1.02	1.02	1.02	1.02
DL-methionine, 99%	0.31	0.31	0.31	0.31	0.31
L-Lysine HCL, 78%	0.25	0.25	0.25	0.25	0.25
L-Threonine	0.09	0.09	0.09	0.09	0.09
L-Valine	0.04	0.04	0.04	0.04	0.04
Vitamin–mineral premix ^1^	0.55	0.55	0.55	0.55	0.55
Sodium chloride	0.38	0.38	0.38	0.38	0.38
OLIVOX^® 2^ mg/kg	0	824	1648	2473	3297
Vitamin E ^3^, mg/kg	40.0	30.0	20.0	10.0	0.0
**Calculated analysis**					
Moisture	12.3	12.3	12.3	12.3	12.3
AME_n_, kcal/kg	2920	2920	2920	2920	2920
Crude protein	21.9	21.9	21.9	21.9	21.9
Ether extract	3.93	3.93	3.93	3.93	3.93
Ash	5.50	5.50	5.50	5.50	5.50
Calcium	0.92	0.92	0.92	0.92	0.92
Digestible phosphorus	0.45	0.45	0.45	0.45	0.45
Sodium	0.16	0.16	0.16	0.16	0.16
Vitamin E, mg/kg	53.7	43.7	33.7	23.7	13.7
**Determined analysis**					
Moisture	12.2	12.5	11.7	11.6	11.8
Gross energy, kcal/kg	3938	3967	3981	3970	3962
Crude protein	22.5	22.7	22.6	22.7	22.5
Ash	5.07	4.70	5.09	4.92	5.08
Vitamin E ^4^, mg/kg	44.7	37.5	26.8	18.2	9.31

^1^ Supplied per kg of diet: vitamin A, 10,000 IU; vitamin D3, 4800 IU; vitamin K3, 3 mg; vitamin B1, 3 mg; vitamin B2, 9 mg; vitamin B6, 4.5 mg; vitamin B12, 40 μg; folic acid, 1.8 mg; niacin, 51 mg; pantothenic acid, 16.5 mg; biotin, 150 μg; Fe as iron (II) sulfate, 54 mg; Cu as copper (II) sulfate, 12 mg; Mn as manganous sulfate, 90 mg; I as potassium iodide, 1.2 mg; Zn as zinc oxide, 66 mg; Se as sodium selenite, 0.18 mg; 6-phytase EC 3.1.3.26, 10,000 FTU; calcium carbonate as a carrier. ^2^ OLIVOX^®^ (60%) in a silica carrier (40%). ^3^ DL-alpha-tocopherol acetate added on top. ^4^ Alpha-tocopherol.

**Table 2:** Ingredient composition and calculated and determined analysis (% as feed basis) of the grower diets (22 to 39 d of age).

	HT0	HT7.5	HT15	HT22.5	HT30
**Ingredients**					
Corn	64.0	64.0	64.0	64.0	64.0
Soybean meal, 47% CP	29.9	29.9	29.9	29.9	29.9
Soy oil	3.05	3.05	3.05	3.05	3.05
Calcium carbonate	0.96	0.96	0.96	0.96	0.96
Monocalcium phosphate	0.50	0.50	0.50	0.50	0.50
DL-methionine, 99%	0.28	0.28	0.28	0.28	0.28
L-Lysine HCL, 78%	0.23	0.23	0.23	0.23	0.23
L-Threonine	0.09	0.09	0.09	0.09	0.09
L-Valine	0.04	0.04	0.04	0.04	0.04
Vitamin–mineral premix ^1^	0.55	0.55	0.55	0.55	0.55
Sodium chloride	0.38	0.38	0.38	0.38	0.38
OLIVOX^® 2^ mg/kg	0	824	1648	2473	3297
Vitamin E ^3^, mg/kg	40.0	30.0	20.0	10.0	0.0
**Calculated analysis**					
Moisture	12.3	12.3	12.3	12.3	12.3
AME_n_, kcal/kg	3100	3100	3100	3100	3100
Crude protein	19.2	19.2	19.2	19.2	19.2
Ether extract	5.87	5.87	5.87	5.87	5.87
Ash	4.65	4.65	4.65	4.65	4.65
Calcium	0.77	0.77	0.77	0.77	0.77
Digestible phosphorus	0.34	0.34	0.34	0.34	0.34
Sodium	0.16	0.16	0.16	0.16	0.16
Vitamin E, mg/kg	54.5	44.5	34.5	24.5	14.5
**Determined analysis**					
Moisture	10.3	11.0	11.1	10.9	11.1
Gross energy, kcal/kg	4136	4103	4088	4117	4093
Crude protein	19.5	19.2	19.3	19.6	19.1
Ash	4.53	4.45	4.42	4.34	4.24
Vitamin E ^4^, mg/kg	48.9	35.7	26.0	19.0	11.3

^1^ Supplied per kg of diet: vitamin A, 10,000 IU; vitamin D3, 4800 IU; vitamin K3, 3 mg; vitamin B1, 3 mg; vitamin B2, 9 mg; vitamin B6, 4.5 mg; vitamin B12, 40 μg; folic acid, 1.8 mg; niacin, 51 mg; pantothenic acid, 16.5 mg; biotin, 150 μg; Fe as iron (II) sulfate, 54 mg; Cu as copper (II) sulfate, 12 mg; Mn as manganous sulfate, 90 mg; I as potassium iodide, 1.2 mg; Zn as zinc oxide, 66 mg; Se as sodium selenite, 0.18 mg; 6-phytase EC 3.1.3.26, 10,000 FTU; calcium carbonate as a carrier. ^2^ OLIVOX^®^ (60%) in a silica carrier (40%). ^3^ DL-alpha-tocopherol acetate added on top. ^4^ Alpha-tocopherol.

**Table 3:** Effects of the replacement of vitamin E (VE) by hydroxytyrosol (HT) in the diet on productive performance of broilers from 0 to 39 days of age.

	HT0	HT7.5	HT15	HT22.5	HT30	SEM ^1^	*p*-Value ^2^		
Hydroxytyrosol, mg/kg	0	7.5	15	22.5	30				
Vitamin E, mg/kg	40	30	20	10	0	(n = 7)	Diet	L ^3^	Q ^4^
**0 to 7 days**									
Body weight 0 d, g	41.2	41.5	41.7	41.2	41.1	0.266	0.474	0.190	0.118
Body weight 7 d, g	189	194	191	195	191	2.010	0.218	0.136	0.175
Average daily gain, g/d	21.1	21.8	21.5	21.9	21.4	0.261	0.182	0.053	0.076
Average daily feed intake, g/d	20.5	20.9	20.6	20.9	20.5	0.260	0.726	0.346	0.328
Feed conversion ratio, g/g	0.973	0.956	0.955	0.947	0.958	0.011	0.605	<0.001	0.002
**8 to 14 days**									
Body weight 14 d, g	524	533	520	525	517	4.388	0.141	0.660	0.408
Average daily gain, g/d	47.8	48.4	47.0	47.3	46.6	0.497	0.117	0.785	0.731
Average daily feed intake, g/d	57.0	57.5	57.0	56.6	55.7	0.624	0.341	0.501	0.228
Feed conversion ratio, g/g	1.192	1.186	1.206	1.198	1.195	0.011	0.833	0.521	0.604
**15 to 21 days**									
Body weight 21 d, g	1095	1100	1078	1096	1091	9.72	0.566	0.499	0.533
Average daily gain, g/d	81.7	81.3	79.8	81.6	81.8	1.06	0.683	0.273	0.231
Average daily feed intake, g/d	105.8	106.0	104.7	104.0	106.3	1.43	0.777	0.430	0.504
Feed conversion ratio, g/g	1.296	1.306	1.297	1.276	1.309	0.011	0.244	0.549	0.564
**22 to 39 days**									
Body weight 39 d, g	3096	3174	3129	3167	3154	38.0	0.594	0.266	0.368
Average daily gain, g/d	111.2	115.3	113.9	115.0	115.1	1.91	0.506	0.213	0.359
Average daily feed intake, g/d	167.5 ^b^	175.8 ^a^	175.4 ^a^	174.0 ^a^	175.0 ^a^	2.04	0.024	0.019	0.055
Feed conversion ratio, g/g	1.508	1.527	1.521	1.513	1.521	0.012	0.783	0.601	0.657
**Global, 0 to 39 days**									
Average daily gain, g/d	78.3	80.5	79.4	80.1	79.8	0.959	0.539	0.271	0.375
Average daily feed intake, g/d	110.2	114.5	113.2	112.6	113.1	1.131	0.125	0.072	0.122
Feed conversion ratio, g/g	1.407	1.422	1.412	1.405	1.417	0.008	0.569	0.900	0.937

^1^ SEM = standard error of the mean. ^2^ Means with a different superscript (^a,b^) in the same row indicate a significant difference between groups (*p* < 0.05). ^3^ L = linear response to the replacement of VE by HT in the diet. ^4^ Q = quadratic response to the replacement of VE by HT in the diet.

**Table 4:** Effects of the replacement of vitamin E (VE) by hydroxytyrosol (HT) in the diet on breast meat quality traits of broilers at 39 days of age.

	Control	HT7.5	HT15	HT22.5	HT30	SEM ^1^	*p*-Value ^2^			
Hydroxytyrosol, mg/kg	0	7.5	15	22.5	30					
Vitamin E, mg/kg	40	30	20	10	0	(n = 7)	Diet	Time ^3^	L ^4^	Q ^5^
**MDA ^6^, mg/kg meat**										
1 day of storage	0.438	0.461	0.406	0.430	0.467	0.024	0.417	-	0.382	0.324
3 days of storage	0.605 ^bc^	0.582 ^c^	0.773 ^ab^	0.640 ^bc^	0.856 ^a^	0.067	0.036	-	0.858	0.592
7 days of storage	0.880 ^b^	0.900 ^b^	1.286 ^ab^	1.242 ^b^	2.354 ^a^	0.206	<0.001	<0.001	0.483	0.038
**Color**										
L* (Lightness)										
1 day of storage	58.1	55.3	56.7	57.0	58.9	0.992	0.121	-	0.077	0.010
3 days of storage	58.5	54.5	57.5	57.1	58.1	1.094	0.105	-	0.235	0.070
7 days of storage	54.0	50.8	53.6	54.0	54.7	1.448	0.361	<0.001	0.272	0.104
a* (Redness)										
1 day of storage	1.52	2.70	2.01	2.40	2.15	0.321	0.126	-	0.116	0.165
3 days of storage	1.68 ^b^	2.96 ^a^	1.65 ^b^	1.82 ^b^	2.10 ^b^	0.267	0.005	-	0.737	0.666
7 days of storage	1.46 ^b^	3.06 ^a^	1.81 ^ab^	1.68 ^ab^	2.07 ^ab^	0.314	0.007	0.483	0.394	0.353
b* (Yellowness)										
1 day of storage	10.1	9.09	8.94	10.1	9.92	0.543	0.387	-	0.226	0.167
3 days of storage	11.4	10.4	9.97	10.9	11.1	0.560	0.418	-	0.088	0.080
7 days of storage	10.8	10.1	10.3	11.2	11.0	0.458	0.361	<0.001	0.538	0.338
**pH**	6.01	6.12	5.87	6.03	5.99	0.074	0.233	-	0.568	0.662
**Drip loss, %**	4.97	3.86	5.27	4.86	4.64	0.631	0.590	-	0.951	0.989

^1^ SEM = standard error of the mean. ^2^ Means with a different superscript (^a,b,c^) in the same row indicate a significant difference between groups (*p* < 0.05). ^3^ Effect of length of storage (1, 3, and 7 days) on MDA concentration and color of the breast. ^4^ L = Linear response to the replacement of VE by HT in the diet. ^5^ Q = Quadratic response to the replacement of VE by HT in the diet. ^6^ MDA = Malonaldehyde concentration.

## Data Availability

The data presented in this study are available on request from the corresponding author.

## References

[1] Estévez M. (2015). Oxidative damage to poultry: From farm to fork. Poult. Sci..

[2] Carvalho R., Shimokomaki M., Estévez M., Petracci M., Berri C. (2017). Poultry meat color and oxidation. Poultry Quality Evaluation.

[3] Khan R.U., Rahman Z.U., Nikousefat Z., Javdani M., Tufarelli V., Dario C., Selvaggi M., Laudadio V. (2012). Immunomodulating effects of vitamin E in broilers. World’s Poult. Sci. J..

[4] National Research Council (NRC) (1994). Nutrient Requirements of Poultry: Ninth Revised Edition.

[5] Hidiroglou N., Cave N., Atwal A.S., Farnworth E.R., McDowell L.R. (1992). Comparative vitamin E requirements and metabolism in livestock. Ann. Rech. Vet..

[6] Santomá G., Mateos G.G., Fundación Española Desarrollo Nutrición Animal (FEDNA) (2018). Necesidades Nutricionales para Avicultura. Normas FEDNA.

[7] Chae B.J., Lohakare J.D., Choi J.Y., Han K.N., Yong J.S., Won H.K., Park Y.H., Hahan T.W. (2005). The efficacy of vitamin E-polyethylene glycol complex on growth performance, chicken meat quality and immunity in broilers. J. Anim. Feed Sci..

[8] Weiss B. How much supplemental vitamins do cows really need?. Proceedings of the 27th Annual Tri-State Dairy Nutrition Conference.

[9] Leeson S. (2007). Vitamin requirements: Is there basis for re-evaluating dietary specifications?. World’s Poult. Sci. J..

[10] Santomá G., Perez de Ayala P. (2022). Evolución de la producción y los programas de alimentación de broilers: Estudio comparativo (2022 vs. 2014). XXXVII Curso de Especialización FEDNA.

[11] Tufarelli V., Passantino L., Zupa R., Crupi P., Laudadio V. (2022). Suitability of dried olive pulp in slow-growing broilers: Performance, meat quality, oxidation products, and intestinal mucosa features. Poult. Sci..

[12] Fernández-Lobato L., García-Ruiz R., Jurado F., Vera D. (2021). Life cycle assessment, C footprint and carbon balance of virgin olive oils production from traditional and intensive olive groves in southern Spain. J. Environ. Manag..

[13] International Olive Council. https://www.internationaloliveoil.org/.

[14] Alburquerque J.A., Gonzálvez J., García D., Cegarra J. (2004). Agrochemical characterisation of “alperujo”, a solid by-product of the two-phase centrifugation mehod for oliveoil extraction. Bioresour. Technol..

[15] Salmoral G., Aldaya M.M., Chico D., Garrido A., Llamas M.R. (2010). The water footprint of olive oil in Spain. Span. J. Agric. Res..

[16] Berbel J., Posadillo A. (2018). Review and analysis of alternatives for the valorisation of agro-industrial olive oil by-products. Sustainability.

[17] Nasopolou C., Zabetaquis I. (2013). Agricultural and aquacultural potential of olive pomace a review. J. Agric. Sci..

[18] Nasopoulou C., Lytoudi K., Zabetakis I. (2018). Evaluation of olive pomace in the production of novel broilers with enhanced in vitro antithrombotic properties. Eur. J. Lipid Sci. Technol..

[19] King A.J., Griffin J.K., Roslan F. (2014). In vivo and in vitro addition of dried olive extract in poultry. J. Agric. Food Chem..

[20] Liehr M., Mereu A., Pastor J.J., Quintela J.C., Staats S., Rimbach G., Ipharraguerre I.R. (2017). Olive oil bioactives protect pigs against experimentally-induced chronic inflammation independently of alterations in gut microbiota. PLoS ONE.

[21] Fernández-Prior M.Á., Fatuarte J.C.P., Oria A.B., Viera-Alcaide I., Fernández-Bolaños J., Rodríguez-Gutiérrez G. (2020). New Liquid Source of Antioxidant Phenolic Compounds in the Olive Oil Industry: Alperujo Water. Foods.

[22] Rigacci S., Stefani M. (2016). Nutraceutical properties of olive oil polyphenols. An itinerary from cultured cells through animal models to humans. Int. J. Mol. Sci..

[23] Rodis P.S., Karathanos V.T., Mantzavinou A. (2002). Partitioning of olive oil antioxidants between oil and water phases. J. Agric. Food Chem..

[24] El-Albbassi A., Kiai H., Hafidi A. (2012). Phenolic profile and antioxidant activities of olive mill wastewater. Food Chem..

[25] Japón-Luján R., Luque de Castro M.D. (2007). Static-dynamic superheated liquid extraction of hydroxytyrosol and other biophenols from alperujo (a semisolid residue of the olive oil industry). J. Agric. Food Chem..

[26] Gerasopoulos K., Stagos D., Kokkas S., Petrotos K., Kantas D., Goulas P., Kouretas D. (2015). Feed supplemented with byproducts from olive oil mill wastewater processing increases antioxidant capacity in broiler chickens. Food Chem. Toxicol..

[27] Herrero-Encinas J., Blanch M., Pastor J.J., Mereu A., Ipharraguerre I.R., Menoyo D. (2019). Effects of bioactive olive pomace extract from *Olea europaea* on growth performance, gut function, and intestinal microbiota in broiler chickens. Poult. Sci..

[28] De la Torre-Carbot K., Jauregui O., Gimeno E., Castellote A.I., Lamuela-Raventós R.M., López-Sabater M.C. (2005). Characterization and quantification of phenolic compounds in olive oils by solid-phase extraction, HPLC-DAD, and HPLC-MS/MS. J. Agric. Food Chem..

[29] Botsoglou N.A., Florou-Paneri P., Christaki E., Fletouris D.J., Spais A.B. (2002). Effect of dietary oregano essential oil on performance of chickens and on iron-reduced lipid oxidation of breast, thigh and abdominal fat tissues. Br. Poult. Sci..

[30] Chen Y.P., Chen X., Zhang H., Zhou Y.M. (2013). Effects of dietary concentrations of methionine on growth performance and oxidative status of broiler chickens with different hatching weight. Br. Poult. Sci..

[31] Balzan S., Cardazzo B., Novelli E., Carraro L., Fontana F., Currò S., Laghetto M., Trocino A., Xiccato G., Taticchi A. (2021). Employment of phenolic compounds from olive vegetation water in broiler chickens: Effects on gut microbiota and on the shelf life of breast fillets. Molecules.

[32] BOE (2013). RD 53/2013, de 21 de octubre por la que se establecen las normas básicas aplicables para la protección de los animales utilizados en experimentación y otros fines científicos, incluyendo la docencia, Spain. Boletín Of. Estado.

[33] Cobb Broiler Management Guide. https://www.cobb-vantress.com.

[34] Branciari R., Galarini R., Giusepponi D., Trabalza-Marinucci M., Forte C., Roila R., Miraglia D., Servili M., Acuti G., Valiani A. (2017). Oxidative status and presence of bioactive compounds in meat from chickens fed polyphenols extracted from olive oil industry waste. Sustainability.

[35] AOAC International (2005). Official Methods of Analysis of AOAC International.

[36] Rey A.I., Daza A., López-Carrasco C., López-Bote C.J. (2006). Quantitative study of the α-and γ-tocopherols accumulation in muscle and backfat from Iberian pigs kept free-range as affected by time of free-range feeding or weight gain. Anim. Sci..

[37] International Olive Council Determination of Biophenols in Olive Oils by HPLC. COI/T.20/Doc.nº.29. 2009/Rev.1 2017. https://www.internationaloliveoil.org/wp-content/uploads/2022/06/Doc.-No-29-REV-2_ENK.pdf.

[38] Capannesi C., Palchetti I., Mascini M., Parenti A. (2000). Electrochemical sensor and biosensor for polyphenols detection in olive oils. Food Chem..

[39] Salih A.M., Smith D.M., Price J.F., Dawson L.E. (1987). Modified extraction 2-thiobarbituric acid method for measuring lipid oxidation in poultry. Poult. Sci..

[40] Littell R.C., Henry P.R., Ammerman C.B. (1998). Statistical analysis of repeated measures data using SAS procedures. J. Anim. Sci..

[41] Pompeu M.A., Cavalcanti L.F.L., Toral F.L.B. (2018). Effect of vitamin E supplementation on growth performance, meat quality, and immune response of male broiler chickens: A meta-analysis. Livest. Sci..

[42] Surai P.F., Kochish I.I., Fisinin V.I., Kidd M.T. (2019). Antioxidant defense systems and oxidative stress in poultry biology: An Update. Antioxidants.

[43] Sarica S., Toptas S. (2014). Effects of dietary oleuropein supplementation on growth performance, serum lipid concentrations and lipid oxidation of Japanese quails. J. Anim. Physiol. Anim. Nutr..

[44] Xie P., Deng Y., Huang L., Zhang C. (2022). Effect of olive leaf (*Olea europaea* L.) extract addition to broiler diets on growth performance, breast meat quality, antioxidant capacity and caecal bacterial populations. Ital. J. Anim. Sci..

[45] Varmaghany S., Rahimi S., Karimi Torshizi M.A., Lotfollahian H., Hassanzadeh M. (2013). Effect of olive leaves on ascites incidence, hematological parameters and growth performance in broilers reared under standard and cold temperature conditions. Anim. Feed Sci. Technol..

[46] Lee K.W., Everts H., Kappert H.J., Frehner M., Losa R., Beynen A.C. (2003). Effects of dietary essential oil components on growth performance, digestive enzymes and lipid metabolism in female broiler chickens. Br. Poult. Sci..

[47] Ghazanfari S., Mohammadi Z., Adib Moradi M. (2015). Effects of coriander essential oil on the performance, blood characteristics, intestinal microbiota and histology of broilers. Braz. J. Poult. Sci..

[48] Brenes A., Roura E. (2010). Essential oils in poultry nutrition: Main effects and modes of action. Anim. Feed. Sci. Technol..

[49] Leskovec J., Levart A., Zgur S., Jordan D., Pirman T., Salobir J., Rezar V. (2018). Effects of olive leaf and marigold extracts on the utilization of nutrients and on bone mineralization using two different oil sources in broilers. J. Poult. Sci..

[50] Branciari R., Ranucci D., Ortenzi R., Roila R., Trabalza-Marinucci M., Servili M., Papa P., Galarini R., Valiani A. (2016). Dietary administration of olive mill wastewater extract reduces *Campylobacter* spp. prevalence in broiler chickens. Sustainability.

[51] Herrero-Encinas J., Huerta A., Blanch M., Pastor J.J., Morais S., Menoyo D. (2023). Impact of Dietary Supplementation of Spice Extracts on Growth Performance, Nutrient Digestibility and Antioxidant Response in Broiler Chickens. Animals.

[52] Ravindran V., Abdollahi M.R. (2021). Nutrition and digestive physiology of the broiler chick: State of the art and outlook. Animals.

[53] Machlin L.J., Bendich A. (1987). Free radical tissue damage: Protective role of antioxidant nutrients. FASEB J..

[54] Tran K., Wong J.T., Lee E., Chan A.C., Choy P.C. (1996). Vitamin E potentiates arachidonate release and phospholipase A2 activity in rat heart myoblastic cells. Biochem. J..

[55] Robles-Almazan M., Pulido-Moran M., Moreno-Fernández J., Ramirez-Tortosa C., Rodríguez-García C., Quiles J.L., Ramirez-Tortosa M. (2018). Hydroxytyrosol: Bioavailability, toxicity, and clinical applications. Food Res. Int..

[56] Salami S.A., Guinguina A., Agboola J.O., Omede A.A., Agbonlahor E.M., Tayyab U. (2016). Review: In vivo and postmortem effects of feed antioxidants in livestock: A review of the implications on authorization of antioxidant feed additives. Animal.

[57] Lourenço C.F., Gago B., Barbosa R.M., de Freitas V., Laranjinha J. (2008). LDL isolated from plasma-loaded red wine procyanidins resist lipid oxidation and tocopherol depletion. J. Agric. Food Chem..

[58] Herrero-Encinas J., Corrales N.L., Sevillano F., Ringseis R., Eder K., Menoyo D. (2023). Replacement of Vitamin E by an Extract from an Olive Oil by-Product, Rich in Hydroxytyrosol, in Broiler Diets: Effects on Liver Traits, Oxidation, Lipid Profile, and Transcriptome. Antioxidants.

[59] Fletcher D.L. (2002). Poultry meat quality. World’s Poult. Sci. J..

[60] Sirri F., Bianchi M., Petracci M., Meluzzi A. (2009). Influence of partial and complete caponization on chicken meat quality. Poult. Sci..

[61] Rhee K.S., Ziprin Y.A. (1987). Lipid oxidation in retail beef, pork and chicken muscles as affected by concentrations of heme pigments and nonheme iron and microsomal enzymic lipid peroxidation activity. J. Food Biochem..

